# Supplementary material for: Necrotic Bone Fluid Suppresses Energy Metabolism of Porcine PBMC-Derived Macrophages In Vitro
Source: Cells. 2025 Aug 14;14(16):1258. doi: 10.3390/cells14161258 (PMC12384577; doi:10.3390/cells14161258)

## Slide 1
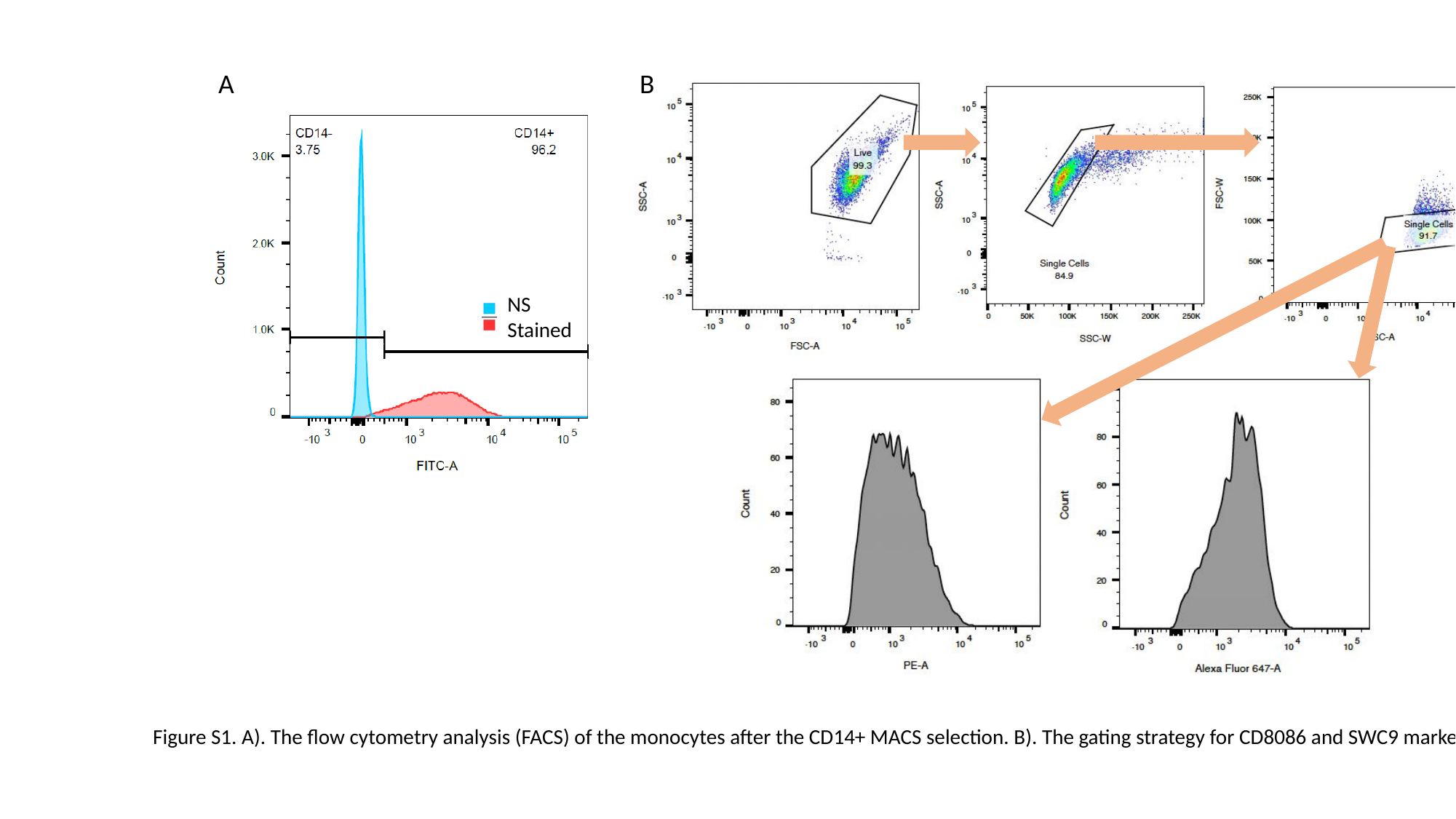

A
B
NS
Stained
Figure S1. A). The flow cytometry analysis (FACS) of the monocytes after the CD14+ MACS selection. B). The gating strategy for CD8086 and SWC9 markers.

## Slide 2
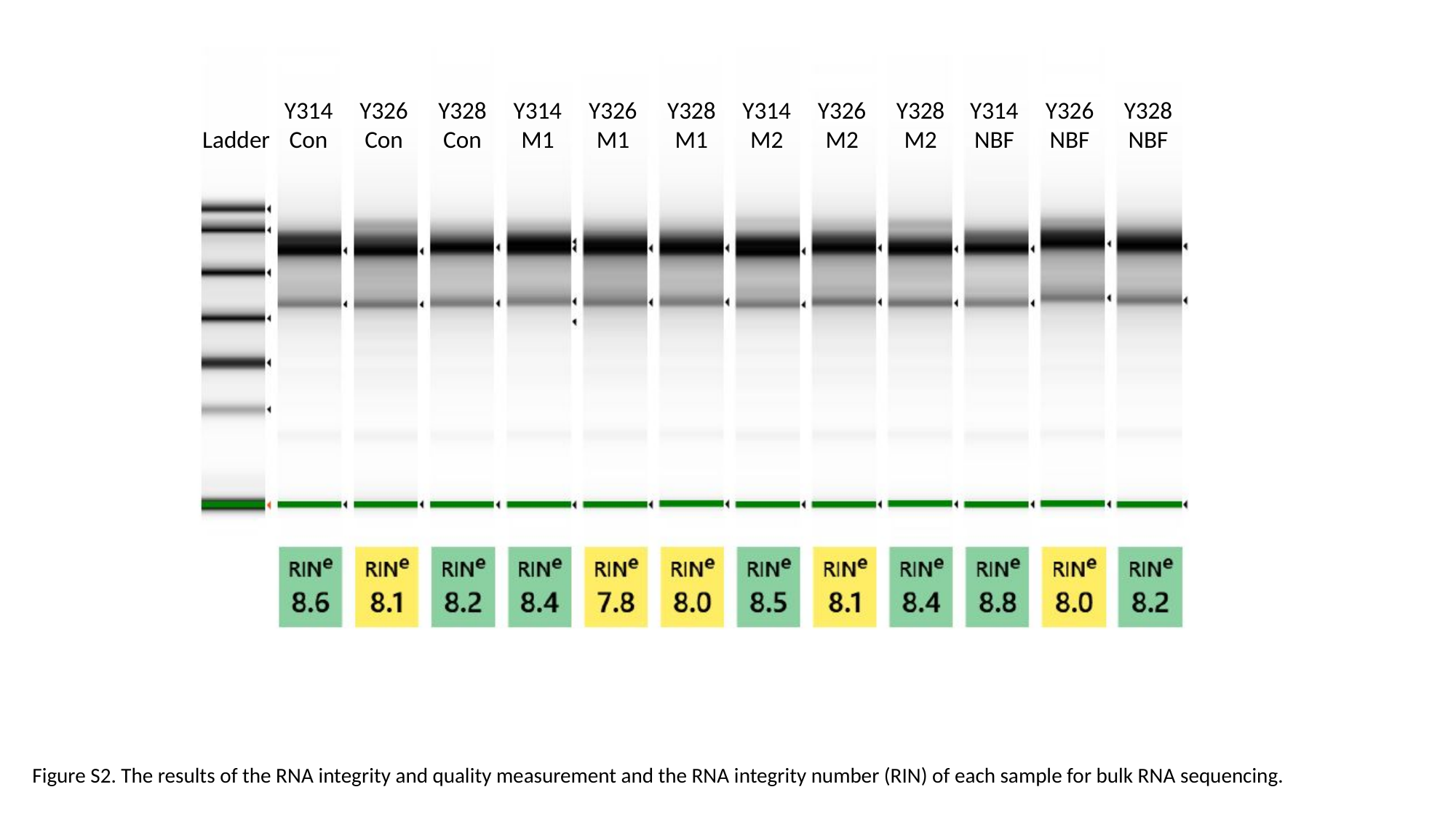

Y314 Con
Y326 Con
Y328 Con
Y314 M1
Y326 M1
Y328 M1
Y314 M2
Y326 M2
Y328 M2
Y314 NBF
Y326 NBF
Y328 NBF
Ladder
Figure S2. The results of the RNA integrity and quality measurement and the RNA integrity number (RIN) of each sample for bulk RNA sequencing.

## Slide 3
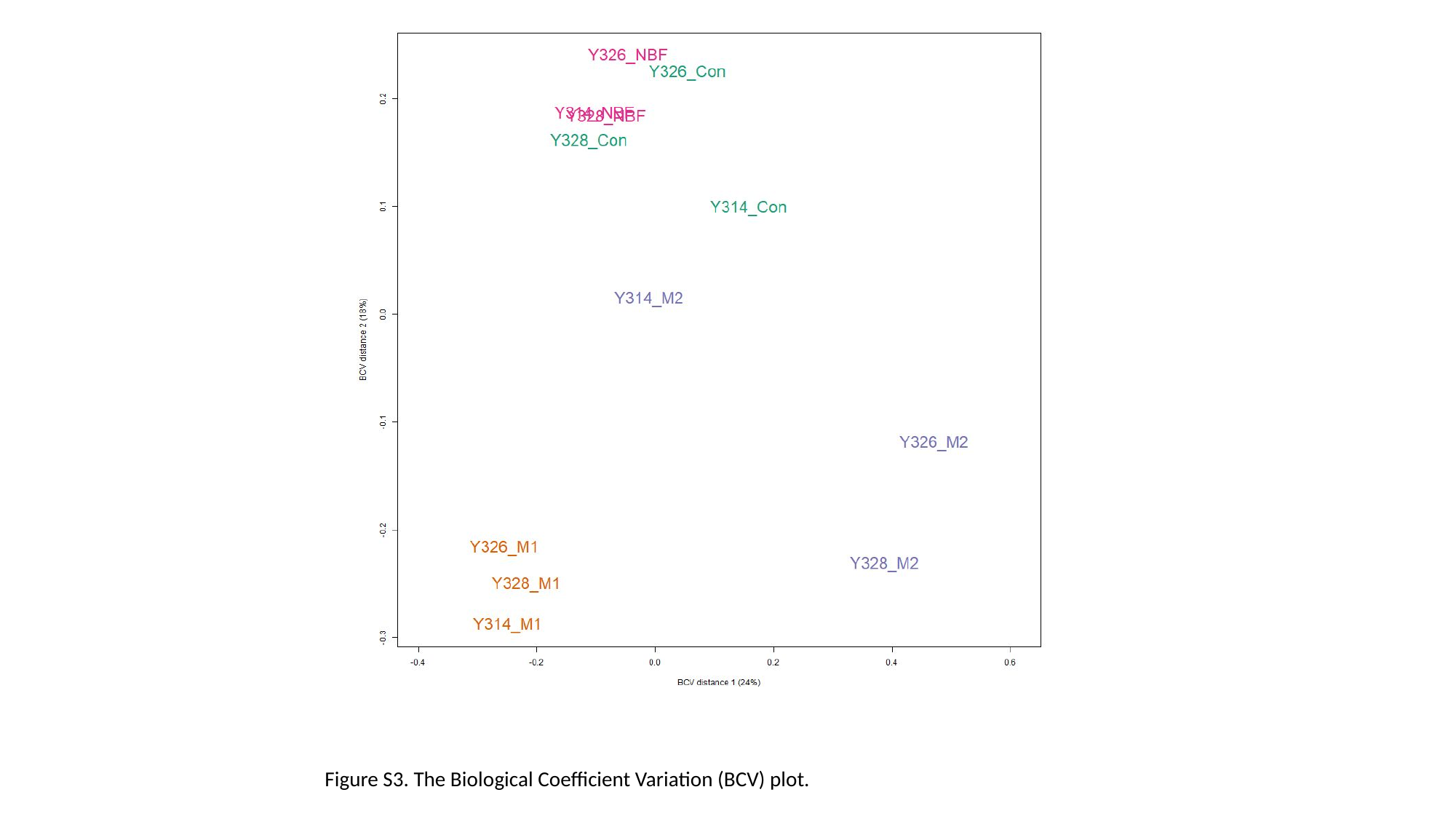

Figure S3. The Biological Coefficient Variation (BCV) plot.

## Slide 4
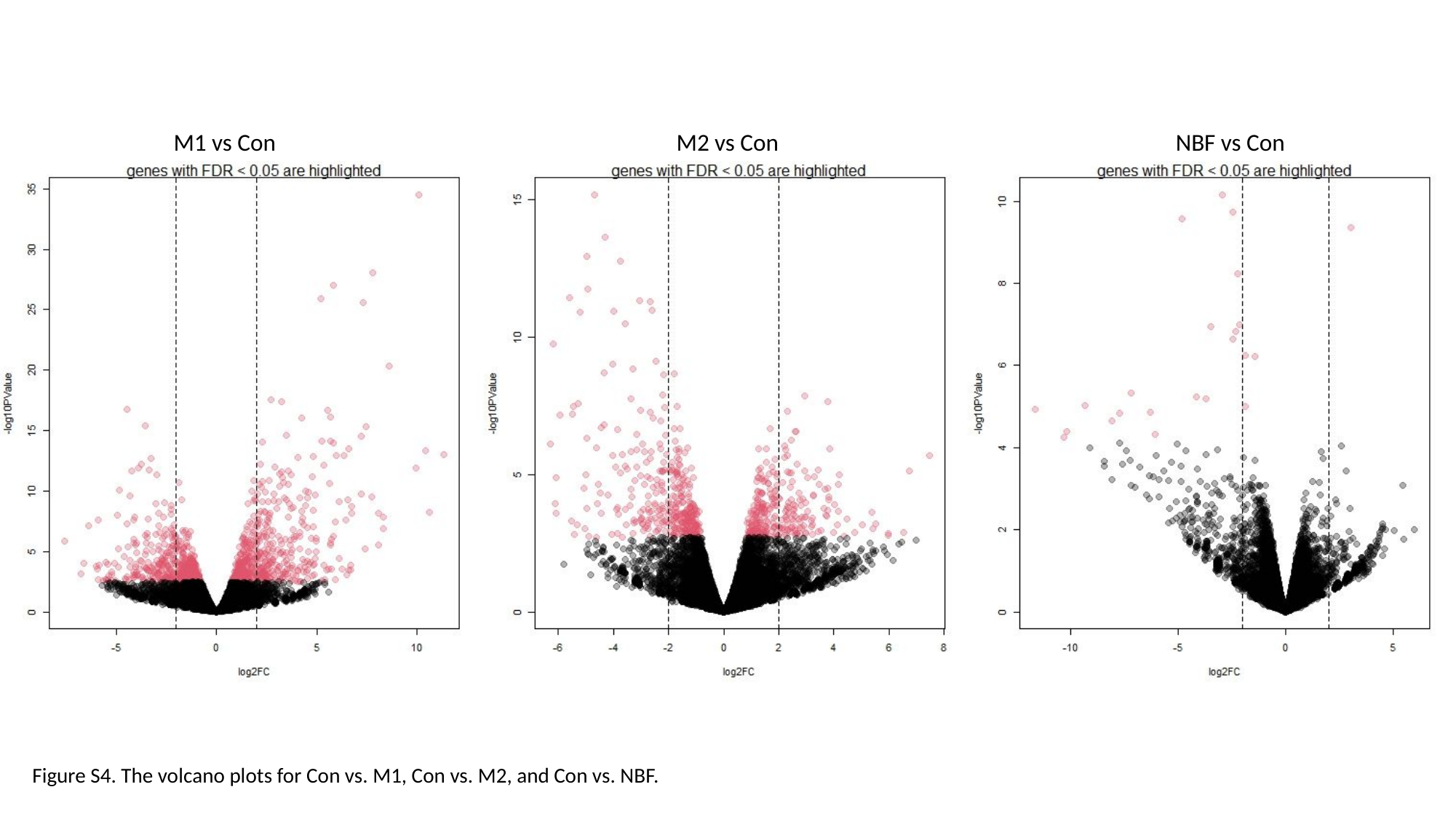

M1 vs Con
M2 vs Con
NBF vs Con
Figure S4. The volcano plots for Con vs. M1, Con vs. M2, and Con vs. NBF.

## Slide 5
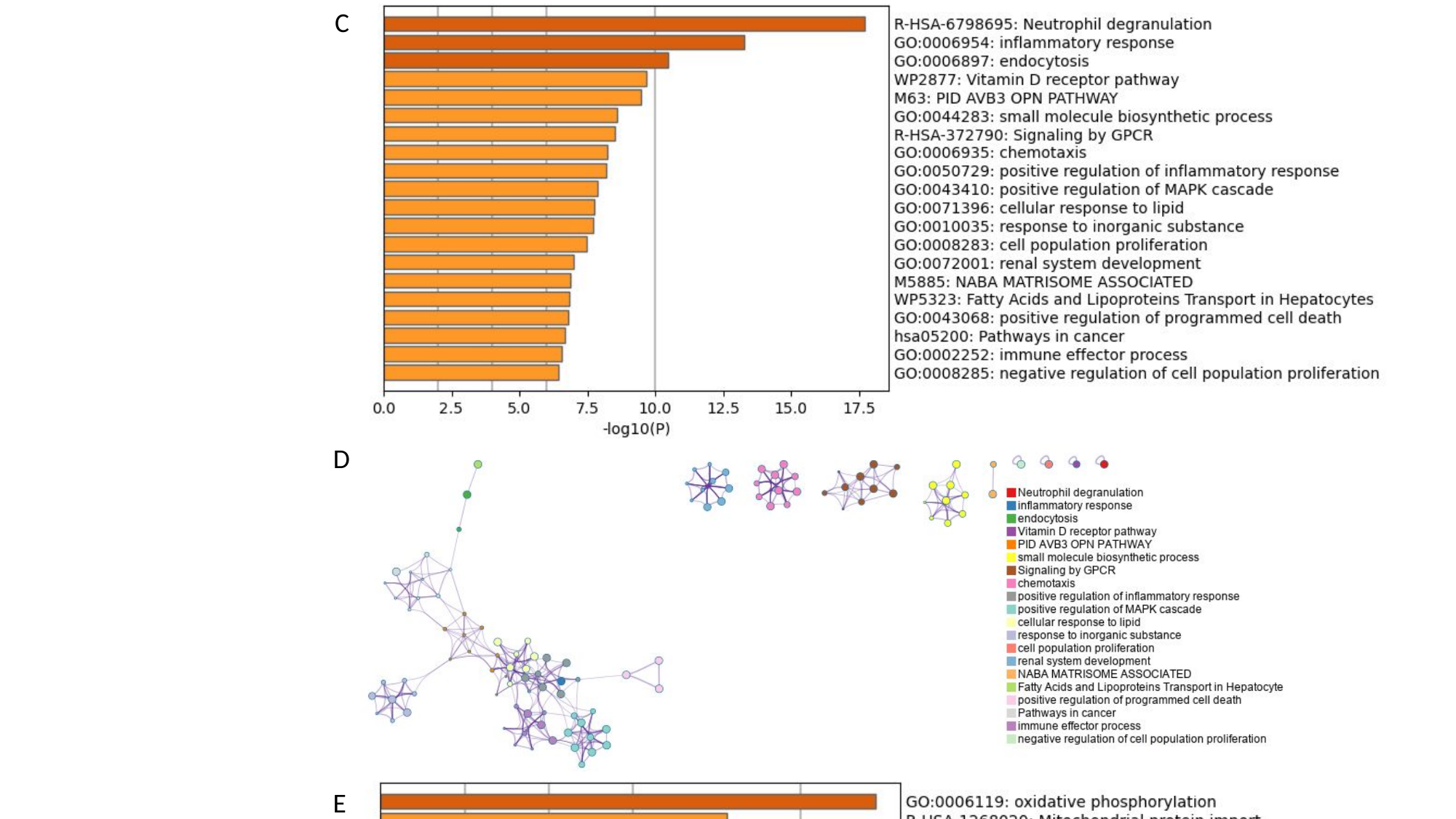

C
A
Con vs M1
B
D
E
F

## Slide 6
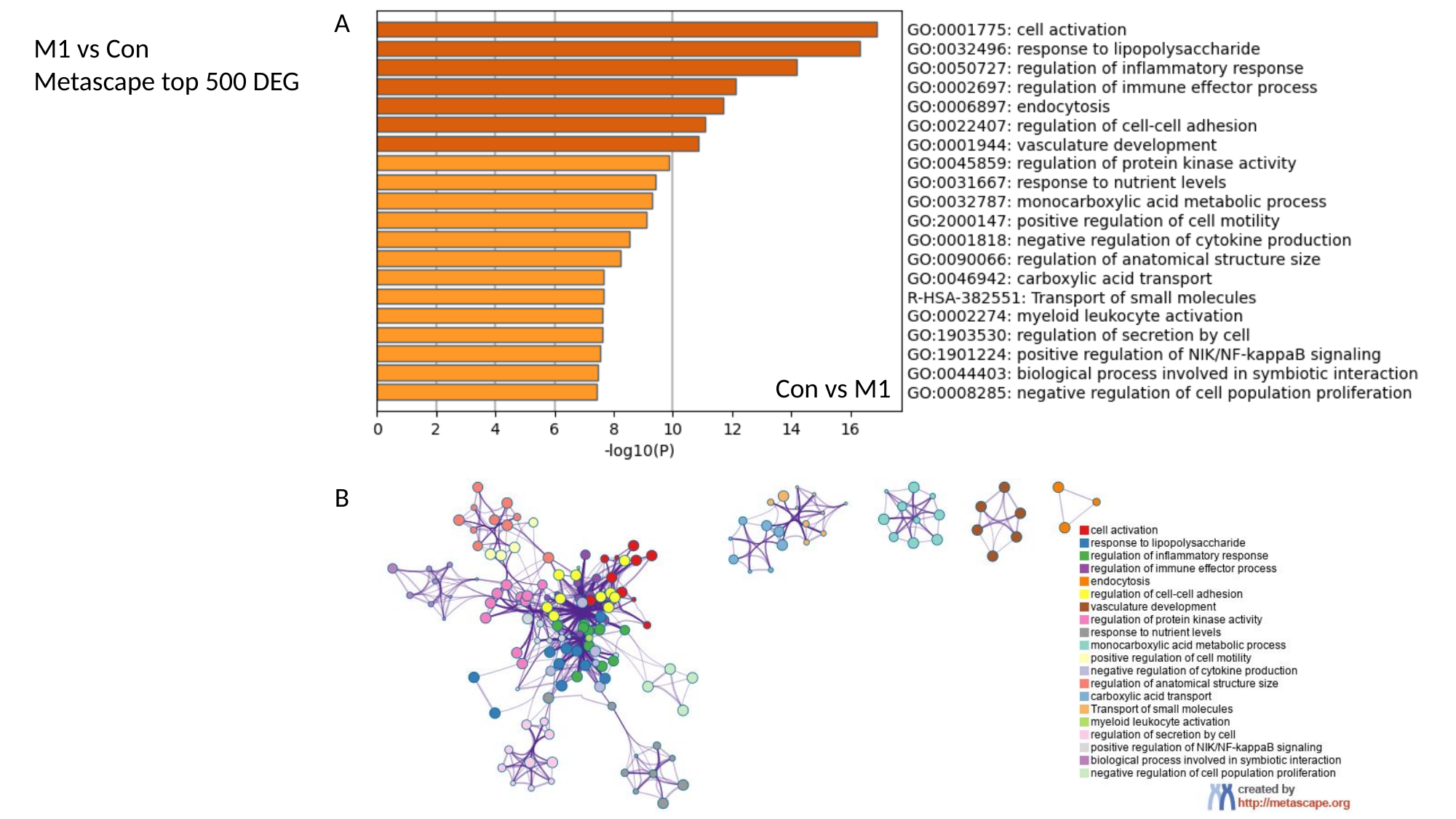

A
M1 vs Con
Metascape top 500 DEG
Con vs M1
B

## Slide 7
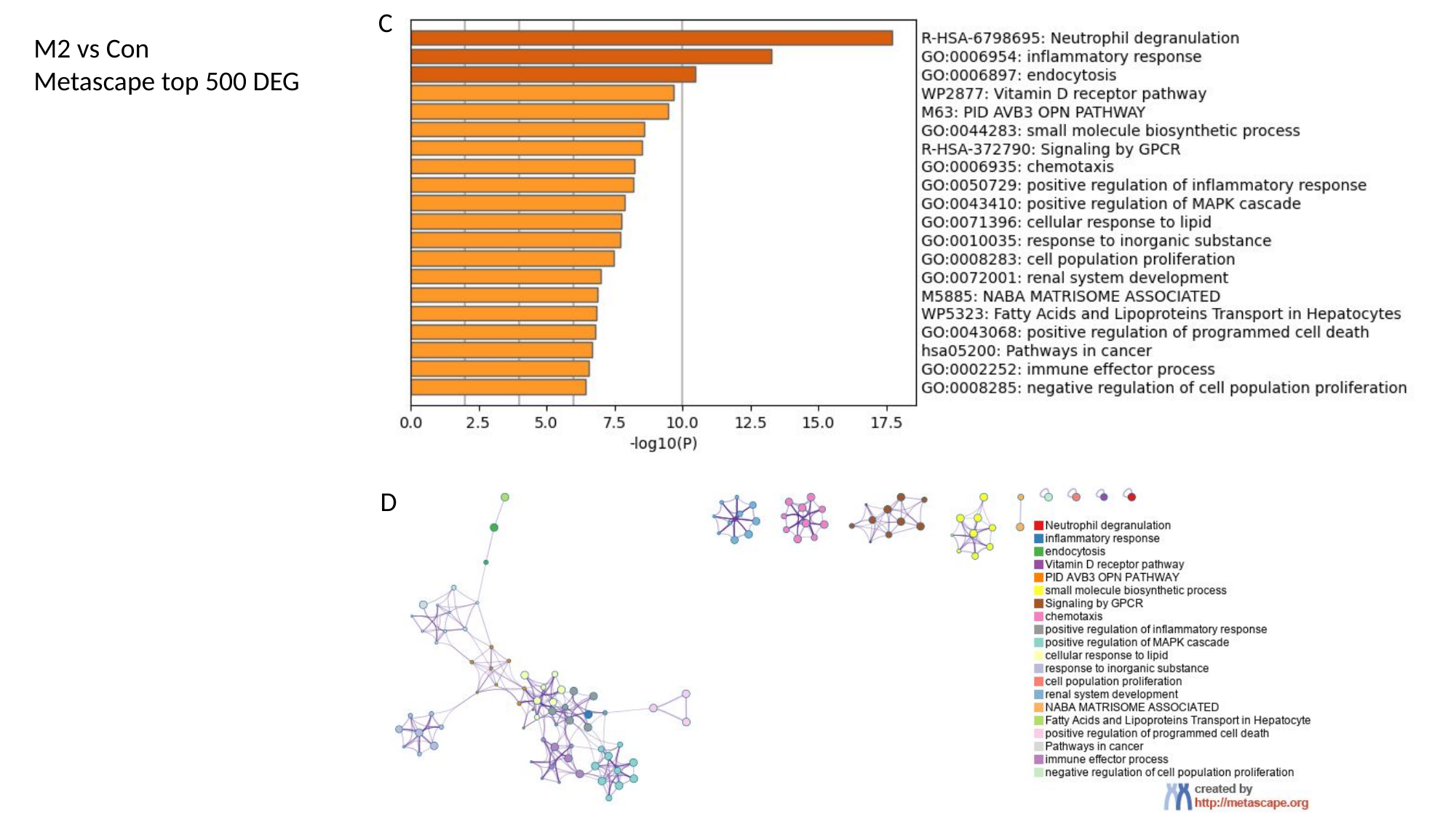

C
M2 vs Con
Metascape top 500 DEG
D

## Slide 8
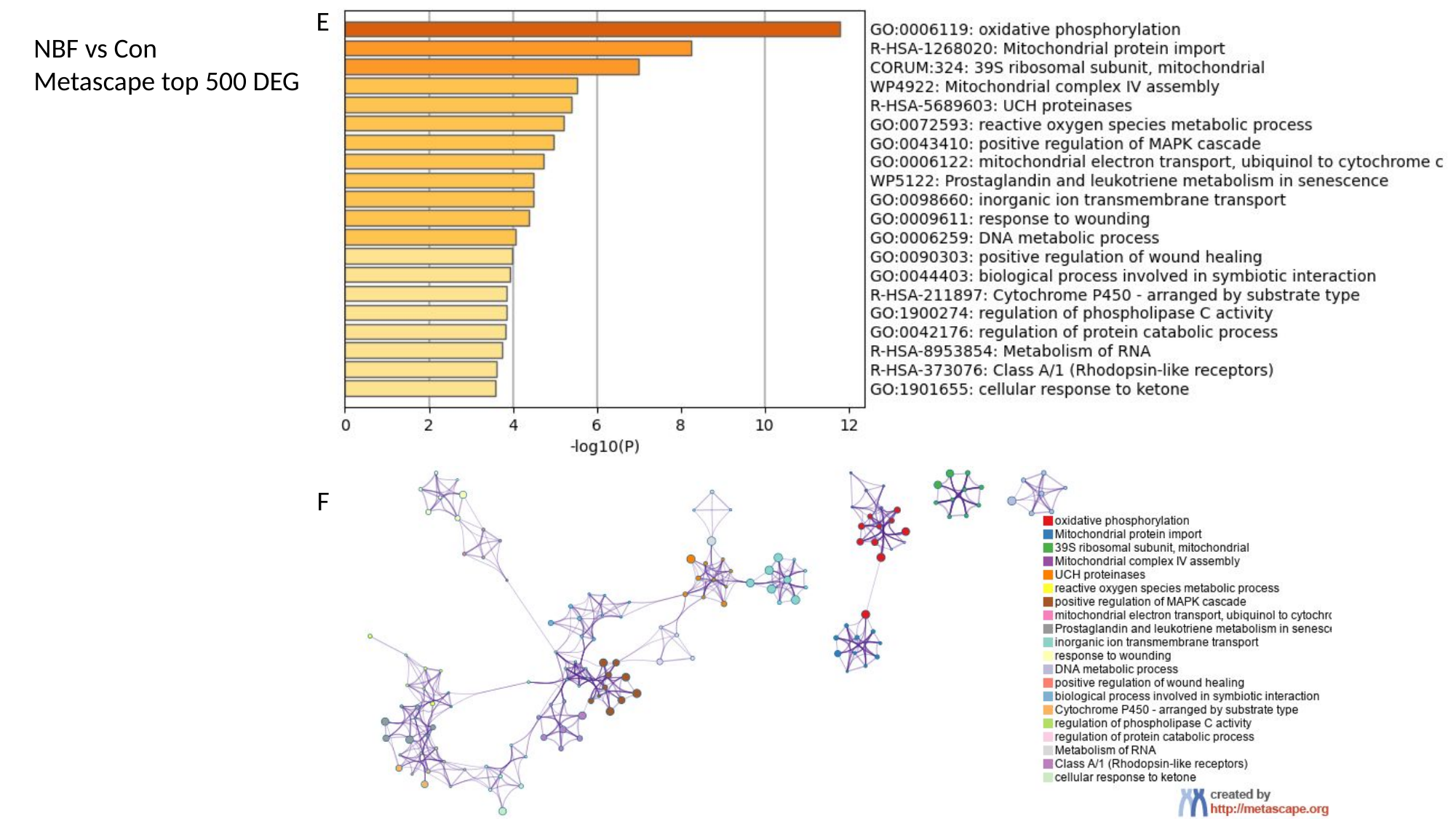

E
NBF vs Con
Metascape top 500 DEG
F

## Slide 9
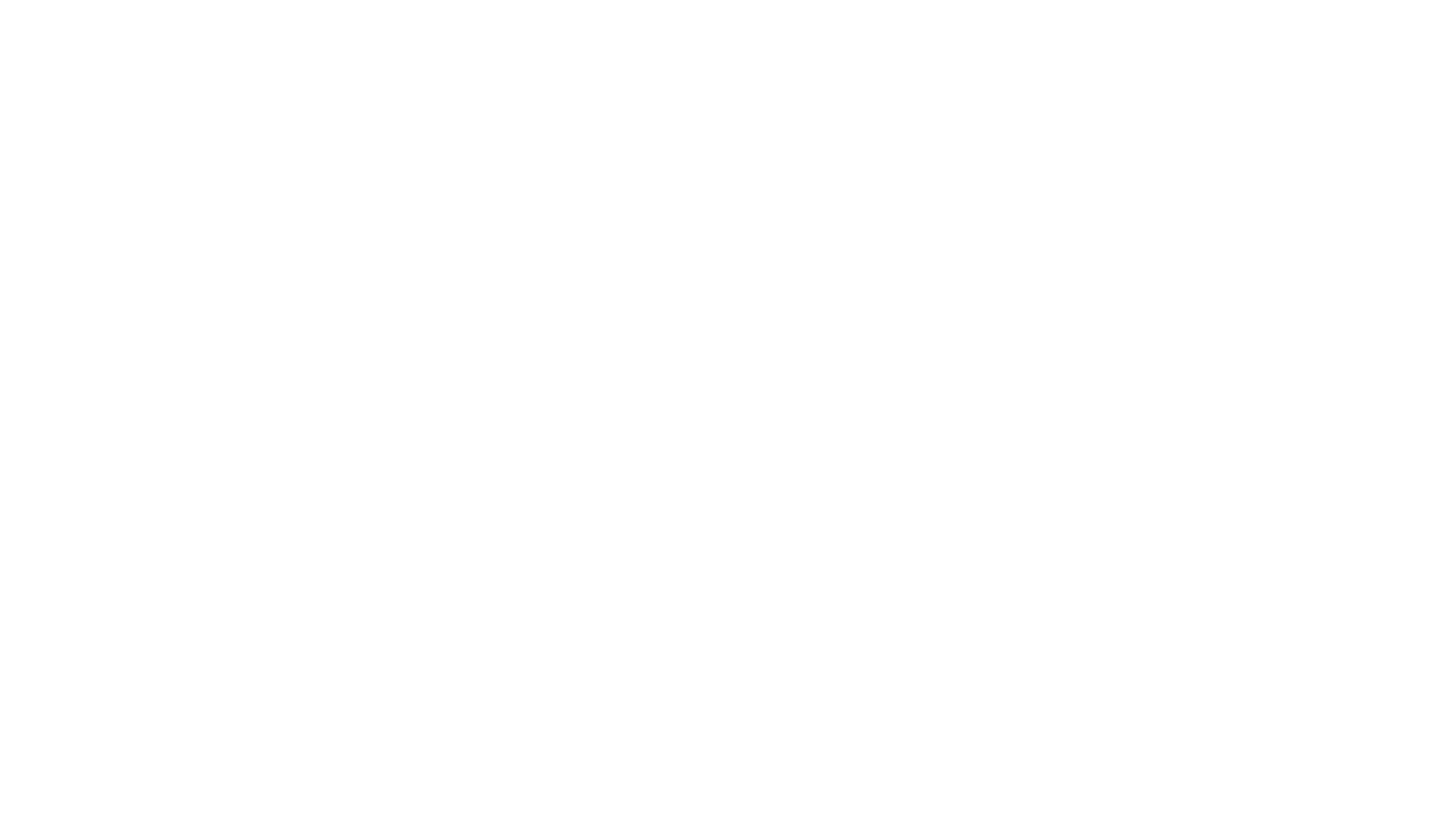

Supplement: Supplementary file 1 [file cells-14-01258-s001.zip › cells-3543395-supplementary.pptx]
